# Association of the Geriatric Nutritional Risk Index and Postoperative Complications in Head and Neck Cancer

**DOI:** 10.1155/jnme/1073981

**Published:** 2025-09-15

**Authors:** Angela A. Colback, Joy Chen, Soroush Ershadifar, Nicole I. Farber, Marianne Abouyared

**Affiliations:** ^1^Department of Otolaryngology-Head and Neck Surgery, University of California, Davis, Sacramento, California, USA; ^2^Department of Otolaryngology-Head and Neck Surgery, Icahn School of Medicine at Mount Sinai, New York, New York, USA

**Keywords:** geriatric nutrition risk index, head and neck cancer, malnutrition

## Abstract

**Background:** Malnutrition is associated with increased morbidity and mortality in patients with head and neck cancer (HNC) undergoing surgery. The ability to identify patients who are malnourished with an objective measure is currently a barrier to prompt screening and interventions. Recognizing the need for a screening tool, we used the geriatric nutritional risk index (GNRI) to assess the prevalence of malnutrition and the association between postoperative complications and GNRI scores.

**Methods:** A retrospective review of medical records was conducted at a tertiary care academic hospital. A total of 49 HNC patients undergoing surgery with a serum albumin obtained within 6 months of surgery were included in this study. GNRI was calculated as follows: (1.487 × serum albumin [g/L] + (41.7 × current weight/ideal body weight [kg]). Analyses were conducted using univariate statistical methods.

**Results:** 49 patients were included in the study, 32 men (65%) and 17 women (35%), with a mean age of 63 + 12 years. Malnutrition was defined by a GNRI score of < 97.5 and was present in 24% of patients (*n* = 12). Malnourished patients had significantly higher rates of postoperative complications and discharge to a skilled nursing facility (SNF) compared to controls.

**Conclusions:** A low GNRI score appears to be a predictor of increased complications after head and neck surgery. The GNRI is a simple tool that requires serum albumin, current body weight, and ideal body weight to objectively assess nutrition status. Further studies are needed to assess the utility of using GNRI to assess malnutrition and identify patients who are at high risk for complications during the postoperative course.

## 1. Introduction

Nutritional depletion is a major consequence of head and neck cancer (HNC) due to challenges with oral intake related to altered anatomy from the tumor itself, side effects with surgery or chemoradiation, and the proinflammatory state underlying the cancer process. Furthermore, lifestyle factors, such as high rates of tobacco and alcohol consumption, in this population contribute to appetite suppression, decreased caloric intake, and alterations in metabolism [1–3]. Inadequate nutrient intake is of clinical concern, as it has been associated with postsurgical complications and decreased overall survival. Specifically, the decline in muscle mass, also known as sarcopenia, has been associated with higher rates of mortality among patients with HNC [2, 4]. Therefore, there is a pressing need to understand effective methods to screen for malnutrition and develop interventions that can be implemented in this unique patient population to improve clinical outcomes.

Timely and accurate diagnosis of malnutrition can be challenging without a universally accepted approach and the limited availability of objective diagnostic markers. Although prealbumin and albumin have traditionally been used, hepatic synthesis is altered in times of inflammation, limiting their use in surgical and oncological patient populations. Rather than using laboratory markers in isolation, alternative methods have been explored, including the prognostic nutritional index (PNI), cross-sectional imaging of skeletal muscle mass, and the nutrition risk index (NRI), among others [5, 6]. The NRI was first used in literature by Buzby et al., and the index combines serum albumin, current body weight, and usual body weight (UBW) to generate a numerical value to evaluate nutritional status [7]. While the NRI is cost-effective and fast to implement, challenges with patients accurately recalling their UBW are a barrier to application in a clinical setting. Thus, the geriatric nutritional risk index (GNRI) was developed as a modification of the NRI to utilize ideal body weight (IBW) rather than UBW [8]. Despite the name, the GNRI does not solely need to be restricted to geriatric patients. Since its inception, the utility of the GNRI has been studied in patients with a wide range of conditions, including heart failure, renal failure, major abdominal surgeries, and various malignancies. Literature shows strong evidence to support that low GNRI scores are associated with higher rates of postoperative complications, poor prognosis, and all-cause mortality in hospitalized patients [9–15].

In the area of HNC, the NRI has been shown to significantly correlate with postoperative complications and 30-day mortality after major surgery [16–18]. However, to our knowledge, there is still a paucity of data regarding the prognostic utility of the GNRI in patients with HNC undergoing surgery. Thus, the purpose of this study is to investigate the prevalence of malnutrition among HNC patients undergoing surgery using the GNRI. In addition, the objective of this study is to stratify patients by GNRI scores and evaluate the ability to use the index as a predictor for complications after surgery.

## 2. Methods

### 2.1. Participants

This is a retrospective review of patients with head and neck mucosal squamous cell carcinoma of the oral cavity, oropharynx, or larynx. Of the 204 patients initially reviewed, 155 (76%) were excluded due to the absence of a serum albumin measurement within six months prior to surgery, as this parameter is essential for GNRI calculation. This exclusion criterion, while limiting sample size, was necessary to ensure the validity of nutritional status assessment and postoperative outcome correlation. Inclusion criteria were as follows: albumin within 6 months prior to surgery, mucosal subsite, and surgical excision as the primary treatment modality. If patients had multiple surgeries, the surgery with an albumin within 6 months before their surgery date was chosen to assess outcomes. One patient had two albumin values in this timeframe prior to surgery, and their second albumin was chosen for this study. Patients included in this study underwent a range of surgical procedures, including partial and total laryngectomy, free flap reconstruction, and neck dissection. Tumor staging was based on the AJCC eighth edition staging system, and this information has been incorporated into the revised Tables 1 and 2. Hypopharyngeal cancer patients were excluded due to their low prevalence in this dataset, which precluded meaningful statistical analysis.

Medical records were reviewed for data abstraction. The data obtained included the following: patient demographics, comorbidities, preoperative labs, tumor subsite, adjuvant therapy, route of nutrition, date of death or last follow-up, and treatment complications. Postoperative complications were defined as adverse events occurring within 30 days of surgery, including infections, fistula, flap failure, and return to the operating room. Postoperative infections were defined as clinical diagnoses requiring antibiotic therapy. The study protocol was reviewed and approved by the UC Davis Medical Center Institutional Review Board, and informed consent was waived due to the retrospective nature of the study. Patient data were anonymized to ensure confidentiality.

**Figure 1:**
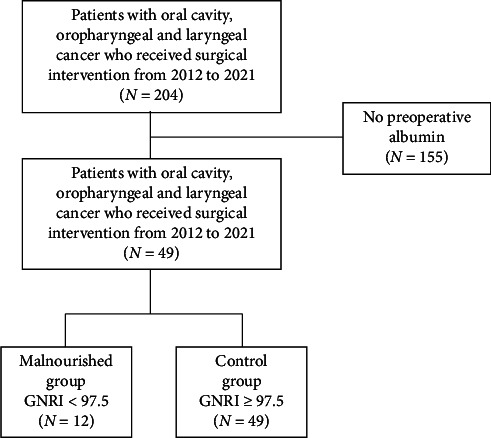
Flowchart of included and excluded patients. Abbreviation: GNRI, geriatric nutritional risk index.

A patient selection algorithm is presented in Figure 1.

### 2.2. Nutritional Assessment by GNRI

Serum albumin and preoperative weight were extracted in a retrospective manner from electronic medical records. The IBW was calculated using the Devine formula for males and females [19]. IBW for men over 60 inches = 50 + (2.3 × [height − 60]) and IBW for women over 60 inches = 45.5 + (2.3 × [height − 60]). The GNRI was calculated as follows: GNRI = (1.487 × serum albumin [g/L]) + (41.7 × current weight/ideal body weight [kg]). We chose a 6-month preoperative window for albumin measurement to ensure adequate sample size while capturing baseline nutritional status. Although shorter intervals may more accurately reflect perioperative conditions, the retrospective nature of this study limited the availability of more recent data. If the preoperative weight exceeded IBW, a score of 1 was assigned to the current weight/IBW ratio for the calculation based on prior studies [8].

The participants were stratified according to the following cutoff values: “high risk: < 83.5,” “moderate risk: 83.5 to < 97.5,” “mild risk: 97.5 to < 100,” and “normal: ≥ 100.” [20, 21].

### 2.3. Statistical Analysis

Statistical analysis was performed using SPSS statistical software, Version 28 (SPSS Inc) and R Studio (Version 2022.07.1 + 554). Between-group differences were assessed using independent *t* tests, as appropriate, for continuous data, and Fisher's exact test for categorical data. Values are reported as mean ± SD, median (range), and odds ratios with 95% confidence intervals (CIs), which were calculated when applicable. All tests were two-tailed, and results were considered statistically significant if *p* < 0.05.

## 3. Results

Two hundred and four charts were reviewed of patients who underwent surgery for cancer of the oral cavity, oropharynx, or larynx between June 2012 and June 2021. One hundred and fifty-five patients (76%) did not have an albumin within six months of surgery and were excluded from the study. Of the 49 patients included in the study, there were 13 patients (27%) with GNRI < 97.5, and they were thus categorized as malnourished. Thirty-six patients (73%) with GNRI ≥ 97.5 were categorized as having normal nutritional status and served as the control for this study.

### 3.1. Distribution and Classification of GNRI

The mean preoperative GNRI in the 49 patients was 104 ± 15. Among this cohort, there were 5 patients (10%) with GNRI < 83.5, 8 patients (16%) with GNRI 83.5–97.5, 0 patients with GNRI 97.5–100, and 36 patients (73%) with GNRI > 100.

### 3.2. Patient Characteristics

Thirty-two males (65%) and seventeen females (35%) were included in this study. The characteristics of all patients are listed in Table 1. The mean age was 63 ± 12 and ranged from 36 to 91 years. The malnourished group did not differ significantly from the normal controls in terms of mean age (mean 63.6 years vs. mean 63.0 years, *p*=0.88), gender (male 75% vs. 62%, *p*=0.5), race (White 92% vs. 62%, *p*=0.21), and enteral feeding prior to surgery (25% vs. 8%, *p*=0.15). In addition, the malnourished group did not have a higher prevalence of comorbidities, as measured by the Charlson Comorbidity Index (CCI) (3.3 vs. 2.8, *p*=0.27) compared to the control group. The mean BMI was notably higher in the control group compared to the malnourished group (mean 27.1 vs. 20.7, *p* ~ 0.001).

Interestingly, the malnourished group had a higher prevalence of laryngeal cancer (66% vs. 11%, *p* < 0.01) and significantly fewer oropharyngeal and oral cavity cancers. Furthermore, the malnourished group had a significantly greater number of patients discharged with a tracheostomy (83% vs. 27%, p<0.001) and continuing to require enteral nutrition as their primary means of nutrition after surgery (58% vs. 14%, *p*=0.004).

### 3.3. Postoperative Complications

A total of 18 patients (37%) had postoperative complications, as detailed in Table 2. Compared to the normal control group, the malnourished group had a significantly higher number of patients who had complications after surgery (67% vs. 27%, *p*=0.02). Among the malnourished group, a greater percentage of patients had bleeding with transfusion, flap-related complications, DVT or PE, and UTI, although the differences between each complication were not significantly different between the two groups. Furthermore, the malnourished group had a greater number of patients who were discharged to a skilled nursing facility (SNF) (33% vs. 5%, *p*=0.03).

While the patients in the malnourished group required return to the operating room more often than those in the control group, the difference was not found to be statistically significant (33% vs. 14%, *p*=0.19). The average length of stay in the malnourished group was longer than the duration of the control group (19 days vs. 12 days, *p*=0.41). Despite this observed difference, it was not statistically significant.

In addition, postoperative complications, return to the operating room, length of stay, and discharge location were further stratified by GNRI scores of < 83.5, 83.5 to < 97.5, 97.5 to < 100, and ≥ 100. The data for each group are found in Table 2. The data show a general trend that the percentage of patients with complications in each group decreases as the GNRI score increases.

## 4. Discussion

In the present study, we evaluated the association of the GNRI and postoperative complications in patients with HNC. While malnutrition is a common consequence of cancer, patients with HNC are especially at risk. In a prospective epidemiological study by Pressoir et al., among 1545 patients with cancer, the prevalence of malnutrition was 45.6% in HNC, exceeding colorectal, gynecological, breast, and hematological malignancies [22]. It is well established that patients have substantial weight loss during the course of treatment, which is especially problematic considering that up to 40% of patients are already malnourished prior to the initiation of treatment [23, 24].

Despite the high burden of malnutrition in this population, identifying a reliable preoperative screening tool to recognize patients who are malnourished and at risk for associated complications continues to be lacking in the literature. The estimated prevalence of malnutrition can vary between 16% and 50% depending on the tool being implemented. [2, 25, 26]. Historically, serum visceral proteins, including prealbumin and albumin, have been used to diagnose malnutrition. However, the activation of the acute phase response can lead to decreased synthesis of prealbumin and albumin, regardless of the nutritional intake. [27–30]. Laboratory markers are appealing as objective measures, however, the current consensus is that they should be used in conjunction with a nutrition focused physical exam. [31]. The benefit of the GNRI is the incorporation of weight as an anthropometric measurement rather than relying on serum albumin alone. Furthermore, the GNRI has also been shown to correlate well with cross-sectional muscle mass at L3 vertebra, which is a well-validated tool for diagnosis of sarcopenia. [32].

To address the gap in objective screening measures in patients with HNC, we analyzed the GNRI scores of 49 patients with HNC undergoing a variety of surgical procedures. In this retrospective study, we found that nearly 25% of patients were malnourished prior to surgery based on their baseline GNRI score of < 97.5. We specifically elected to use the GNRI rather than the NRI due to the key difference in the GNRI using the “ideal” body weight rather than the “usual” body weight. We considered calculating IBW via the Devine formula to be more objective than relying on patients' reports of their “usual” body weight.

We found that among the malnourished group, there was a significantly greater number of patients with laryngeal cancer compared to the control. One explanation for this observed difference is the variation in swallowing function depending on the primary cancer site. In a retrospective study by Stenson et al., among the 79 patients with Stage III and Stage IV HNC, the primary tumor site was associated with significant differences in swallowing dysfunction. Patients with hypopharyngeal and laryngeal disease had higher rates of esophageal and pharyngeal impairment. In addition, aspiration status was positive in 14% of patients with oral cavity cancer compared to 30% in oropharyngeal and 67% in laryngeal cancer [33]. The observed differences were statistically significant. Not only did we observe more patients with laryngeal cancer in the malnourished group, but this group also had a higher dependence on enteral nutrition prior to surgery (25% vs. 8%, *p*=0.15), although the difference was not statistically significant. Thus, it is plausible that there may be a connection between the location of the tumor site and impaired swallowing function, contributing to decreased oral intake and subsequently malnutrition.

In addition, compared to the control, the malnourished group had more patients with at least one complication after surgery. This finding was statistically significant and consistent with findings that have been documented in the literature [16]. Cancer creates a state of chronic inflammation punctuated by moments of higher levels of physiological stress, such as surgery, contributing to muscle catabolism [34]. Consequently, patients with HNC have increased caloric needs and at least a 120% increase in protein requirement from baseline [35, 36]. Inadequate nutrition intake to meet the elevated metabolic demand leads to catabolism and impairs proper wound healing. In a retrospective study by Vandersteen et al., among the 423 patients who underwent head and neck free flap reconstruction, malnutrition was significantly associated with a higher risk of fistula formation and wound infection [37]. Loss of skeletal muscle mass is especially detrimental as studies have shown that sarcopenia decreases anti-inflammatory mediators, exacerbating muscle breakdown [38].

Among patients with low GNRI scores, one approach to improving outcomes is the use of specific nutrients that have been shown to attenuate inflammation and augment wound healing. In a study by Wu et al., supplemental glutamine was shown to improve albumin in surgical cancer patients, and the authors theorized that it was in part due to the amelioration of stress-related inflammation [39]. Supplementation of arginine has also shown promise due to its role in T-cell proliferation and endothelial protective effects among patients who have vascular injury induced by radiation therapy [40]. In particular, there has been an increasing focus on the role of immunonutrition oral supplementation, as it supplies a combination of immune-modulating nutrients, including arginine, omega-3 fatty acids, and RNA, to promote healing and recovery after surgery [41, 42]. Our findings that laryngeal cancer patients in particular were more likely to be in the low GNRI/malnourished group serve as an important motivator to begin nutritional prehabilitation efforts on this patient population, specifically.

### 4.1. Limitations

There are limitations to this study that must be acknowledged. The retrospective nature of this study limited the inclusion of only patients who had an available serum albumin within 6 months prior to surgery. Since albumin was not routinely checked preoperatively, the number of patients included in the study was reduced, and the small sample size likely limited the ability to detect significant differences between the two groups. This limitation also contributed to wide CIs in our analyses, reflecting increased variability and limiting the precision of our estimates. Future studies with larger cohorts are needed to validate these findings with greater statistical power. Furthermore, errors in classifying postoperative complications and lack of follow-up among patients are both possible sources of bias in this study as well.

The small and clinically heterogeneous sample in this study reflects the diversity of HNC patients, which may influence the generalizability of our findings. Future studies with larger, more uniform cohorts are needed to validate these results. In addition, due to the small sample size, subgroup analyses by cancer subsite (e.g., oral vs. laryngeal) were not performed. We acknowledge that each subsite presents unique perioperative risk profiles that may influence outcomes differently. Future studies with larger patient populations should include stratified analyses to capture these differences.

In addition, there was a significantly greater number of patients in the malnourished group who had a free flap reconstruction compared to the control group. This imbalance between the two groups can be a possible confounder, as the need for a free flap may indicate a larger tumor size, which can contribute to worse swallowing dysfunction, malnutrition, and lower GNRI scores prior to surgery. Given the retrospective nature of this study and the limited sample size, we opted not to perform propensity score matching, as this would further reduce statistical power. Potential imbalances between groups, such as differences in the prevalence of total laryngectomy, may have influenced the results. In addition, we did not include albumin and BMI as covariates in the logistic regression model due to their collinearity with GNRI. Similarly, “discharged with tracheostomy” and ”feeding modality after surgery” were not included as covariates as they are postoperative outcomes. These factors should be accounted for in future studies with larger sample sizes and more advanced statistical approaches to minimize confounding. Furthermore, free flap reconstructions are more extensive procedures, thus often requiring more wound care needs, leading to a longer length of stay, and are prone to more complications assessed in this study. It is important to take into consideration this confounder when drawing conclusions regarding the prognostic utility of the GNRI in clinical practice.

Data on caloric and protein intake were not collected routinely in these patients and thus were unavailable for this retrospective study. The lack of dietary intake data limits our ability to fully assess the relationship between nutrition and postoperative outcomes. Future studies should include detailed dietary intake to better assess the impact of preoperative nutritional status.

## 5. Conclusion

This retrospective study found that nearly 25% of patients with HNC are malnourished prior to surgical treatment, as defined by a low GNRI. The GNRI is a time-effective, objective approach to detecting malnutrition. Our study found that low GNRI scores significantly led to increased postoperative complications, return to the operating room, increased length of stay, and discharge to a SNF. The GNRI is a promising tool that is straightforward to implement and should be further studied as it is proving to be a promising tool to assess surgical risks associated with malnutrition in a clinical setting.

## Figures and Tables

**Table 1:** Baseline demographics and clinical characteristics.

	GNRI < 97.5, *N* = 12	GNRI ≥ 97.5, *N* = 37	*p* value	OR (95% CI)
Mean age, year (mean ± SD)	63 + 13	63.6 + 12	0.88	NA
Gender			0.532	
Male (%)	9 (75)	23 (62)		1 [reference]
Female (%)	3 (25)	14 (38)		0.55 (0.08–2.74)
Mean BMI, kg/m^2^ (mean ± SD)	20.7 ± 4.4	27.1 + 6	0.005	
< 18.5 (%)	3 (25)	0 (0)		0.0 (0.0–1.53)
≥ 18.5 to < 25.0 (%)	8(67)	16 (43)		1 [reference]
25.0 to 30.0 (%)	1 (8)	12 (32)		0.17 (0.0–1.6)
≥ 30.0 (%)	0 (0)	9 (24)		0.0 (0.0–1.37)
Albumin (mean ± SD)	3.0 ± 0.6	4.0 ± 0.3	< 0.001	NA
Race			0.718	
White (%)	11 (79)	23 (66)		1 [reference]
Black (%)	0 (0)	2 (6)		NA
Other (%)	3 (21)	10 (29)		2.0 (0.36–11.23)
Primary site			0.002	
Oral cavity (%)	1 (8)	21 (57)		1 [reference]
Oropharynx (%)	2 (17)	11 (30)		3.8 (0.31–46.9)
Larynx (%)	9 (75)	5 (13)		37.8 (3.85–371.3)
Flap reconstruction			0.021	
No (%)	3 (25)	22 (59)		1 [reference]
Yes (%)	9 (75)	15 (41)		0.23 (0.03–1.14)
Discharged with tracheostomy			0.001	
No (%)	2 (17)	27 (73)		1 [reference]
Yes (%)	10 (83)	10 (27)		12.68 (2.18–139.02)
Feeding modality before surgery			0.194	
Oral (%)	9 (75)	34 (89)		1 [reference]
NG or PEG tube (%)	3 (25)	4 (11)		3.11 (0.5–18.62)
Feeding modality after surgery			0.004	
Oral (%)	5 (42)	32 (86)		1 [reference]
NG or PEG tube (%)	7 (58)	5 (14)		8.42 (1.62–50.95)

*Note:* Odds ratios (ORs) and 95% confidence intervals (CIs) were calculated using logistic regression analysis, comparing each characteristic against a reference category. An OR greater than 1 indicates a higher likelihood of the outcome occurring in the malnourished group (GNRI < 97.5), whereas an OR less than 1 suggests a lower likelihood. Confidence intervals provide a range within which the true OR is expected to fall; wider intervals reflect greater variability and potential uncertainty due to small sample size. For example, an OR of 8.42 (95% CI: 1.62–50.95) for “feeding modality after surgery” indicates that malnourished patients were significantly more likely to require enteral nutrition postoperatively. Tumor staging was based on the AJCC eighth edition, with stages included in the analysis to account for variations in tumor burden and surgical complexity. BMI, body mass index (calculated weight in kilograms divided by height in meters squared); NG, nasogastric.

Abbreviation: PEG, percutaneous endoscopic gastrostomy.

**Table 2:** Postoperative complications.

	GNRI < 97.5, *N* = 12	GNRI ≥ 97.5 *N* = 37	*p* value	OR (95% CI)
Number of patients with complications^a^	8 (67)	10 (27)	0.02	5.19 (1.1–29.18)
Bleeding with transfusion (%)	2 (17)	1 (3)	0.14	6.83 (0.33–434.76)
Flap congestion (%)	1 (8)	1 (3)	0.43	3.17 (0.04–263.25)
Flap necrosis (%)	2 (11)	4 (17)	0.63	1.63 (0.13–13.5)
Fistula (%)	3 (25)	1 (3)	0.04	11.21 (0.80–643.82)
Hematoma (%)	0 (0)	1 (3)	1	0 (0–120)
Infection (%)	0 (0)	1 (8)	0.25	Inf (0.08–Inf)
Dehiscence (%)	1 (8)	0 (0)	0.43	3.18 (0.04–263.25)
DVT or PE (%)	1 (8)	0 (0)	0.25	Inf (0.08–Inf)
Pneumonia (%)	0 (0)	2 (5)	1	0 (0–16.76)
UTI (%)	1 (8)	1 (3)	0.43	3.18 (0.03–263.25)
Return to the OR				
No (%)	8 (67)	32 (86)		1 [reference]
Yes (%)	4 (33)	5 (14)	0.195	3.11 (0.49–18.62)
Total LOS, day (mean ± SD)	19 ± 23	12 ± 34	0.41	NA
Discharge location^b^				
Home (%)	8 (67)	34 (92)		1 [reference]
SNF (%)	4 (33)	2 (5)	0.02	8.5 (1.31–54.82)
AMA (%)	0 (0)	1 (3)	0.9	0.001 (4.48e − 48, 5.39e41)

Abbreviations: AMA, against medical advice; DVT, deep vein thrombosis; LOS, length of stay; OR, operating room; PE, pulmonary embolism; SNF, skilled nursing facility; UTI, urinary tract infection.

^a^More than 1 complication possible per patient.

^b^For categorical variables with more than two levels, such as discharge location, *p* values were calculated for each category separately against the reference group.

## Data Availability

The datasets generated and/or analyzed during the current study are available from the corresponding author upon reasonable request.
